# Effects of Cold Exposure on Some Physiological, Productive, and Metabolic Variables in Lactating Dairy Goats

**DOI:** 10.3390/ani10122383

**Published:** 2020-12-11

**Authors:** Wellington Coloma-García, Nabil Mehaba, Xavier Such, Gerardo Caja, Ahmed A. K. Salama

**Affiliations:** 1 Facultad de Medicina Veterinaria, Universidad Agraria del Ecuador (UAE), Guayaquil 090114, Ecuador; wncg_100583@hotmail.com; 2 Tests and Trials Ltd., Ignacio Luzán, 24, 22400 Monzón, Spain; nabil@testsandtrials.com; 3 Grupo de Investigación de Rumiantes (G2R), Departamento de Ciencia Animal y de los Alimentos, Universitat Autònoma de Barcelona, 08193 Barcelona, Spain; xavier.such@uab.cat (X.S.); gerardo.caja@uab.cat (G.C.)

**Keywords:** milk production, metabolism, cold environment, dairy goats

## Abstract

**Simple Summary:**

In the current study the impact of cold temperatures (CT; −3 to 6 °C) on milk production and metabolism was evaluated in dairy goats. Compared to goats in thermoneutral conditions (TN; 15 to 20 °C), CT goats produced lower amounts of milk, but their milk contained more fat and protein. Consequently, the yield of energy-corrected milk did not vary between TN and CT goats. Additionally, feed intake did not vary between treatments. The CT goats mobilized body fat reserves to spare glucose and cover the increased needs for heat production under low temperatures. In conclusion, CT goats produced lower milk yield, but their milk contained greater fat and protein compared to TN goats. Furthermore, cold temperatures induced metabolic changes that included body fat mobilization without changes in blood insulin values.

**Abstract:**

Low winter temperatures in some regions have a negative impact on animal performance, behavior, and welfare. The objective of this study was to evaluate some physiological, metabolic, and lactational responses of dairy goats exposed to cold temperatures for 3 weeks. Eight Murciano-Granadina dairy goats (41.8 kg body weight, 70 days in milk, and 2.13 kg/day milk) were used from mid-January to mid-March. Goats were divided into 2 balanced groups and used in a crossover design with 2 treatments in 2 periods (21 days each, 14 days adaptation and 7 days for measurements). After the first period, goats were switched to the opposite treatment. The treatments included 2 different controlled climatic conditions with different temperature-humidity index (THI) values. The treatments were: thermoneutral conditions (TN; 15 to 20 °C, 45% humidity, THI = 58 to 65), and cold temperature (CT; −3 to 6 °C, 63% humidity, THI = 33 to 46). Goats were fed ad libitum a total mixed ration (70% forage and 30% concentrate) and water was freely available. Goats were milked at 0800 and 1700 h. Dry matter intake, water consumption, rectal temperature, and respiratory rate were recorded daily (days 15 to 21). Body weight was recorded at the start and end of each period. Milk samples for composition were collected on 2 consecutive days (days 20 and 21). Insulin, glucose, non-esterified fatty acids (NEFA), ß-hydroxybutyrate (BHB), cholesterol, and triglycerides were measured in blood on d 21. Compared to TN goats, CT goats had similar feed intake, but lower water consumption (−22 ± 3%), respiratory rate (−5 ± 0.8 breaths/min), and rectal temperature (−0.71 ± 0.26 °C). Milk yield decreased by 13 ± 3% in CT goats, but their milk contained more fat (+13 ± 4%) and protein (+14 ± 5%), and consequently the energy-corrected milk did not vary between TN and CT goats. The CT goats lost 0.64 kg of body weight, whereas TN goats gained 2.54 kg in 21 days. Blood insulin and cholesterol levels were not affected by CT. However, values of blood glucose, NEFA, hematocrit, and hemoglobin increased or tended to increase by CT, whereas BHB and triglycerides decreased. Overall, CT goats produced less but concentrated milk compared to TN goats. Despite similar feed intake and blood insulin levels CT goats had increased blood glucose and NEFA levels. The tendency of increased blood NEFA indicates that CT goats mobilized body fat reserves to cover the extra energy needed for heat production under cold conditions.

## 1. Introduction

Exposure to hot or cold environments negatively affects production, reproduction, welfare, and health of ruminants [1,2]. Some regions in the world experience high ambient temperatures during the summer and very low temperatures during the winter. This wide difference in temperatures between seasons represents a challenge for animals in these regions to cope with conditions in the summer and winter [2]. Ruminants can cope with cold temperatures by physiological and behavioral changes that allow them to maintain their energy homeostasis [3]. Energy requirements for maintenance have been reported to increase by 20% under cold temperatures and can double if the animal is wet and exposed to the wind [4]. The increment in maintenance requirements results in fewer available nutrients for production, and, consequently, decreased performance and production efficiency.

Previous studies focused on cows exposed to cold temperatures, which reported an increase in feed intake [5] and heat production [6], a decline in milk yield [7], an immune depression [8], and a damage of the peripheral tissue, such as ears and frostbite teats [9]. In Manchega dairy ewes, Ramon et al. [10] showed that milk yield fluctuations during the winter (0.9 and 12.3 °C, for minimum and maximum temperatures, respectively) are of a greater magnitude than during the summer (15.5 and 32.2 °C for minimum and maximum temperatures, respectively). Furthermore, Peana et al. [11] found that milk yield decreased by 25% in sheep when temperatures were below 0 to 3 °C compared to 15 to 18 °C.

Goats are commonly described as rustic and adaptable animals with a wide range of thermal tolerance [12]. Therefore, goats are spread worldwide and raised under different production systems. However, research argues that goats are adversely affected by cold temperatures with different extents of thermal tolerance depending on the breed [13]. Compared to other species, especially cattle, few detailed studies have been carried out to evaluate the impact of low ambient temperatures on dairy goats. In fact, most of the studies published 30 to 40 years ago in Saanen [14,15] and Angora goats [16] tested the effects of cold temperatures over a short-term period (2 to 3 days), and little is known on relatively long-term effects, especially on metabolic indicators under controlled environmental conditions.

In addition, thermoregulatory mechanisms and the value range of thermal comfort are not well defined in goats. The lower border of the thermo-neutral zone is known as the lower critical temperature (LCT), below which the animal increases heat production to keep the homeothermy. In fact, there is a high uncertainty in determining the LCT value in goats. Magee [17] showed that the heat production rate (Cal/h) is increased when ambient temperature is below 13 °C in dry, pregnant goats. In another study [18], the LCT of castrated male feral goats fed at the maintenance level has been shown to be 9 °C. Furthermore, adult goats are supposed to have an LCT as low as 0 °C [19].

Our hypothesis was that exposing dairy goats to cold temperatures (−3 to 6 °C) for prolonged time (i.e., 3 weeks) in controlled climatic conditions would reduce milk production and induce changes in the energetic metabolism. The objective of the present study was to evaluate the effect of cold ambient temperatures on some physiological responses, milk production, and blood metabolite profile in Murciano-Granadina dairy goats. Murciano-Granadina is a subtropical breed and one of the most important dairy breeds in Spain and the Mediterranean region [20]. These goats have been exported to several countries all over the world because of their rusticity and good milk composition. However, no studies have been conducted to evaluate the response of Murciano-Granadina goats to cold temperatures under controlled conditions.

## 2. Materials and Methods

### 2.1. Animals

The Ethical Committee of Animal and Human Experimentation of the Universitat Autonoma de Barcelona (UAB) approved the management practices and animal care procedures used in the current study (ref. 3142). This approval followed the instructions described in the Spanish (R.D. 53/2013) and EU (Council Directive: 2010/63) legislations.

Eight multiparous (parity number = 3.4 ± 0.4) lactating Murciano-Granadina dairy goats with healthy and symmetrical udders from the herd of the experimental farm of the UAB were enrolled in the current study from mid-January to mid-March. At the start of the experiment, goats were, on average, 41.8 ± 0.8 kg body weight (BW), 2.13 ± 0.14 L/d milk, and 70 ± 1 days of lactation. Goats were blocked in two balanced groups according to BW, milk yield, and days in milk. Throughout the experiment, goats were individually kept in sawdust-bedded pens (1.0 × 1.5 m) equipped with individual feeders and drinkers. Since there are no studies reported in the literature examining the impact of cold temperatures in Murciano-Granadina goats, power analysis was performed based on data reported in heat-stressed goats of the same breed and use the same experimental design and facilities [21,22]. The fat-corrected milk data recorded for control and heat stress goats (2.10 vs. 1.78, SD = 0.22, respectively) were used to calculate the anticipated number of goats needed to provide sufficient statistical power. A sample size of eight goats per treatment was calculated using a level of significance of *p* < 0.05 and 80% power to detect differences if present.

### 2.2. Experimental Design and Treatments

Goats were exposed to two environmental conditions varying in the temperature-humidity index (THI). The experimental design was a crossover with two treatments in two periods, lasting 21 days, and four goats each. Each experimental period consisted of 14 days for adaptation and 7 days for sample collection and measurements (days 15 to 21). Goats were switched to the opposite treatment in the second period. Treatments were: thermoneutral conditions (TN, 15 to 20 °C and 45% relative humidity, THI = 58 to 65), and cold temperatures (CT, −3 to 6 °C and 63% relative humidity, THI = 33 to 46). The change in temperatures throughout the day for CT was gradual (0.75 °C/h) with minimum and maximum temperatures at 1:00 a.m. and 1:00 p.m., respectively. Considering 0 °C as the LCT in adult goats [19], our goats remained for 8 h under this presumable limit of cold stress. The photoperiod was maintained constant at 12 h light: 12 h dark (8:00 a.m. to 8:00 p.m.).

### 2.3. Management Conditions

Before the experiment, goats were kept indoors and managed with the remaining herd as one group in the same barn. Goats were sheltered in a barn enclosed by three walls, with the other open to the west and with windbreakers. The roof was thermo-isolated and provided with stack chimneys and fans. During the four weeks before the experiment (mid-December to mid-January), barn temperature ranged from 10.5 to 17.8 °C (averaged 12.8 ± 0.8 °C). After moving to the experimental rooms, goats had a two-week pre-experimental period in control conditions to adapt to the experimental facilities.

During the experiment, both TN and CT goats were kept indoor in two adjacent rooms in the same building. In each room, there were four identical separated sawdust-bedded pens (1.0 × 1.5 m) equipped with individual feeders and drinkers. The four pens were next to each other and animals were able to see each other. Feed was offered in individual plastic feed boxes (54 × 34 × 31 cm) hanged outside in one wall. A stainless-steel drinker was provided with a float valve and connected by a hose to a 20-L plastic tank. For the TN room (4 × 8 × 4 m), ambient temperature was controlled by electric heaters provided with a thermostat (3.5 kW, General Electric, Barcelona, Spain) that turned on when ambient temperature was below 15 °C and turned off when temperature was 20 °C. The temperature in the CT room (4 × 6 × 2.3 m) was controlled by a refrigeration system (Model STL200Z012, Rivacold SRL, Vallefoglia, Italy) and programmable relay (Logo, Siemens, Munich, Germany). The environmental temperature and humidity were recorded every 10 min throughout the experiment by data loggers (Opus 10, Lufft, Fellbach, Germany). The THI values were calculated according to the National Research Council guidelines (NRC) [23].

Feed was provided individually ad libitum as a total mixed ration consisting of 70% alfalfa hay and 30% concentrate (soybean hulls, 45.0%, barley whole-grain, 10.0%, gluten feed, 10.0%, rapeseed meal, 10.0%, cracked oat grain, 5.0%, cracked corn grain, 4.0%, soybean meal, 44%, soybean oil, 5.0%, dicalcium phosphate, 2.5%, sugarcane molasses, 2.0%, salt, 0.5%, mineral-vitamin mix, 1.0%). The diet was formulated to cover nutrient requirements according to the recommendations of the French National Institute for Agricultural Research (INRA) [24]. The chemical composition and nutritive value of the ration is shown in Table 1. Clean water and mineral blocks were freely available for each goat (composition: Na, 36.74%, Ca, 0.32%, Mg, 1.09%, Zn, 5 g/kg, Mn, 1.50 g/kg, S, 0.912, g/kg, Fe, 0.304 g/kg, I, 75 mg/kg, Co, 50 mg/kg, and Se, 25 mg/kg, Ovi Bloc, Sal Cupido, Terrassa, Spain). Diet was offered twice a day in two portions at 9:30 AM and 9:30 PM. Feed refusals were collected and weighed at 9:00 a.m., and the amount of feed offered was adjusted to allow a 15% to 20% refusals. The actual refusal level averaged 19.0% and 19.6% (SEM = 0.5%) for TN and CT goats, respectively.

Goats were milked twice daily at 8:00 a.m. and 5:00 p.m. using a portable milking machine (Westfalia Separator Ibérica S.A, Granollers, Spain) set at 40 kPa, 90 pulses/min, and a 66% pulsation ratio. The milking routine included cluster attachment without teat cleaning or udder preparation, machine milking, machine stripping, and teat dipping in an iodine solution (P3-io shield, Ecolab Hispano-Portuguesa, Barcelona, Spain).

### 2.4. Sampling and Data Collection

#### 2.4.1. Rectal Temperature and Respiratory Rate

Rectal temperatures and respiratory rates were recorded daily (days 15 to 21) at 8:00 a.m., 12:00 p.m., and 5:00 p.m. The rectal temperature was measured with a digital veterinary thermometer (model “Accu-vet, ST714AC”, Tecnovet, Barcelona, Spain, range of 32 to 42 °C, and accuracy ±0.10 °C). The respiratory rate was recorded as the number of breaths per minute by counting the flank movements with the help of a stopwatch.

#### 2.4.2. Feed Intake and Water Consumption

Feed intake and water consumption were recorded daily (days 15 to 21) using a digital scale (model Fv-60K, A&D Mercury PTY, Thebarton, Australia, accuracy ±20 g). Feed intake and water consumption were determined by the difference between the weight of the amount offered and the amount refused. Three feed samples were collected and pooled at the beginning of each period and stored at 4 °C for composition analysis. Feed samples were ground through a 1-mm stainless steel screen, and then analyzed for dry matter (DM), acid detergent fiber, neutral detergent fiber, and ash contents, according to analytical standard methods [25]. The Dumas method [25] with a Leco analyzer (Leco Corp., St. Joseph, MI, USA) was used for N determinations and crude protein was calculated as percentage of N × 6.25.

#### 2.4.3. Body Weight and Energy Balance Calculation

Goats were weighed in two consecutive days at the start (days 1 and 2) and end (days 20 and 21) of each experimental period to measure the change in BW. Body weight was recorded after the AM milking and before feeding using a digital scale (Tru-Test AG500 Digital Indicator, Auckland, New Zealand, accuracy ±20 g). Values of BW were also used to calculate the net energy balance (EB) using the following equation:

EB = net energy intake − (NE_M_ + NE_L_). (1)

Net energy for maintenance was calculated using the following equation [24]:

NE_M_ = (0.0406 × BW ^0.75^, INRA). (2)

Energy maintenance requirements were increased by 20% for CT goats as recommended by NRC [4]. Net energy for lactation was calculated by using the following equation [24]:

NE_L_ = [0.389 + 0.0052 (fat, g/kg−35) + 0.0029 × (protein, g/kg−31)] × milk yield (INRA). (3)

#### 2.4.4. Milk Yield and Milk Composition

Milk yield of individual goats was recorded daily at each milking during the measurement period. Milk samples for composition were collected at days 20 and 21 and composition data were averaged. Milk samples were composited in proportion to milk yield at each milking and stored at 4 °C with a preservative (bronopol tablet, D&F Control System, San Ramon, CA, USA). Milk samples were analyzed by the Laboratori Interprofessional Lleter de Catalunya (Allic, Cabrils, Barcelona, Spain). The analyses included fat, protein (N × 6.38), and lactose using MilkoScan (MilkoScan FT2–infrared milk analyzer, Foss 260, DK-3400 Hillerød, Denmark). For milk somatic cell count (SCC), an automatic cell counter (Fossomatic 5000, Foss Electric, Hillerød, Denmark) was used. All devices were calibrated for goat milk.

#### 2.4.5. Blood Insulin and Metabolites

Blood samples were collected at day 21 of each period at 8:00 a.m. before the morning feeding and milking from the jugular vein into a 10-mL tube with spray-coated sodium heparin and a 10-mL tube with spray-coated K2-EDTA (BD Diagnostics, Franklin Lakes, NJ, USA). Plasma was obtained by centrifugation for 15 min at 1500× *g* and 4 °C and kept at −30 °C until the analysis of insulin, glucose, non-esterified fatty acids (NEFA), ß-hydroxybutyrate (BHB), triglycerides, and cholesterol, according to the manufacturer’s instructions. Insulin concentrations were determined using an ELISA kit (Mercodia Ovine Insulin ELISA, Mercodia, AD Bioinstruments, Barcelona, Spain). Glucose was measured by the hexokinase method using an automatic analyzer system (Olympus AU400, Hamburg, Germany). The NEFA were determined by the colorimetric enzymatic test ACS-ACOD method using a commercial kit (Wako Chemicals, Neuss, Germany). The BHB was determined by a kinetic enzymatic method using a commercial kit (Ranbut, Randox, UK). Triglycerides were analyzed with an enzymatic method (glycerol phosphate oxidase), and cholesterol was also analyzed by an enzymatic method (cholesterol esterase/peroxidase).

Blood was also sampled (roughly 0.3 mL) at 8:00 a.m. for the immediate analysis of some major ions and metabolites. A drop of blood was placed into disposable cartridges (i-STAT EC8+, Abbott Point of Care Inc., Princeton, NJ, USA). Then, the cartridge was inserted into an i-STAT hand-held analyzer, and the results of urea, Na, K, Cl, total CO_2_ concentration, anion gap, hematocrit, hemoglobin, pH, partial pressure of CO_2_ (pCO_2_), HCO_3_^−^, and base excess were obtained.

### 2.5. Statistical Analyses

Data were analyzed by the Mixed procedure of SAS version 9.1.3 (SAS Institute Inc., Cary, NC, USA) for repeated measurements. The statistical mixed model contained the fixed effects of the treatment (CT vs. TN), day (1 to 7), and period (1 and 2), the random effect of the animal, the interactions treatment × day, treatment × period, and the residual error. For rectal temperature and respiratory rate values measured at 8:00 a.m., 12:00 a.m., and 5:00 p.m., a fixed effect of the measurement hour was added to the model. The autoregressive (1) covariance matrix was used based on the fit statistics of SAS. For data collected only once, there were no repeated measurements, and, consequently, the day effect was excluded from the model. Differences between least squares means were determined with the PDIFF option of SAS. Data were presented as least squares means and the standard error of the difference (SED). Significance was declared at *p* < 0.05 unless otherwise indicated.

## 3. Results and Discussion

### 3.1. Rectal Temperature and Respiratory Rate

As shown in Table 2, rectal temperatures at 8:00 a.m. and 12:00 p.m. as well as the daily average values were lower in CT goats than TN. However, at 5:00 p.m., when the ambient temperature reached its highest level for CT (6 °C), the rectal temperature was similar for both TN and CT goats. Rectal temperatures increased from 8:00 a.m. to 5:00 p.m. by 0.88 °C in CT goats in accordance with the increment in the ambient temperature throughout the day. The highest rectal temperature in TN goats was also observed at 5:00 p.m. Studies on crossbred (Corriedale × Suffolk) sheep indicated that exposure to cold temperatures (2 °C) results in decreased rectal temperatures [26]. Similar results were obtained after 6 h of exposure to 4.5 °C in Coopworth × Texel sheep [3]. However, with milder cold ambient temperatures (i.e., 9 °C), Barnett et al. [27] did not detect changes in a rectal temperature of Merino castrated rams. In the current experiment, when CT goats were at 6 °C, they were able to keep the rectal temperature value (38.58 °C) similar to the range of rectal temperatures (38.64 to 38.82 °C) recorded for TN goats kept at 15 to 20 °C.

The respiratory rate was lower (−5 breaths/min on average) in CT goats compared to TN throughout the experimental period (Table 2). This agrees with the results of Sano et al. [26] in crossbred sheep exposed to a cold environment (2 °C). It is well documented that the respiratory rate increases dramatically in heat-stressed dairy goats to dissipate heat by evaporation [12,28]. The decreased respiratory rate in the present study could be part of acclimation to cold temperatures by which goats decrease evaporative heat loss through the respiratory tract. No interaction between treatment and the measurement day (*p* > 020) was detected for a rectal temperature or respiratory rate data.

### 3.2. Feed Intake, Water Consumption, and Body Weight Change

Dry matter intake did not vary (*p* > 0.05) between TN and CT goats (Table 3). In accordance with our results, Thompson and Thompson [14] in a short-term experiment (2 days) detected no effect of cold ambient temperature (0.5 to 1 °C with or without blown air) on feed intake of Saanen goats. Additionally, Bøe and Ehrlenbruch [13] reported that inclement cold weather had no effect on time spent feeding in goats. Sheep exposed to 2 °C [26] or 9 °C [27] have similar dry matter intake to those animals on 20 to 26 °C. Nevertheless, studies in cattle showed that animals exposed to cold temperatures significantly increase their feed intake [5].

In the present study, it is possible that the DM intake was at its maximum capacity since the ration contained 70% roughages, and goats were not able to eat more. In fact, rumen volume has been reported to decrease in cold temperatures [29]. Another explanation for the no change in DM intake is that the severity of cold (i.e., −3 to 6 °C) in the current study was not enough to induce a change in DM intake. More research is warranted to test different intensities of cold and their interaction with concentrate: forage ratio in the diet.

Water consumption and water: DM intake ratio were decreased (*p* < 0.05) by 22% and 16%, respectively, in CT goats compared to TN goats (Table 3). Similarly, water consumption was reduced by 46% to 62% in Saanen goats exposed to 0.5 to 1 °C compared to 22 °C [14]. The respiratory rate decreased (Table 2) and, consequently, water evaporation declined, which might partially explain the reduced need for water consumption. No interaction between treatment and measurement day (*p* > 0.20) was detected for feed intake or water consumption data.

Despite the similar feed intake, TN goats gained BW (2.54 kg) at the end of the experimental period, whereas CT goats lost 0.64 kg (Table 3, *p* < 0.01). This result might indicate that CT goats needed more energy to produce heat, and they mobilized body fat reserves, as shown hereafter by the increased blood NEFA. The calculated EB was positive in both TN and LT goats (Table 3) but was lower (*p* < 0.01) in CT than TN goats. The fact that CT goats lost BW even though they presumably were in slightly positive EB, which might indicate that the 20% increment used in the calculation of their maintenance requirements as recommended [4] was not enough to cover the needs. Scibilia et al. [30] reported a 32% increase in the maintenance energy requirement for calves raised at −4 °C compared to calves housed at 10 °C.

### 3.3. Milk Yield and Milk Composition

The effects of cold temperatures on milk yield and composition are shown in Table 3. Average values of milk yield decreased by 13% (*p* < 0.001) in CT compared to TN goats. Similarly, Thompson and Thompson [14] and Faulkner et al. [15] observed a decline in milk secretion and milk yield of Saanen dairy goats exposed to cold temperatures (−0.5 to 1 °C) for one to two days. Brouček et al. [7] also reported decreases of 2 kg in milk yield of cows when the temperature was continuously −10 °C for two months. In contrast to our results, McBride and Christopherson [31] reported that milk yield in Suffolk-cross shorn ewes is not affected by cold ambient temperatures (0 °C) for eight weeks. Discrepancy among results could be related to differences in species and the breed of animals used in each study. No interaction between treatment and measurement day (*p* > 0.20) was detected for milk yield.

The decreased milk yield in CT goats could be a consequence of an increment in maintenance requirements by the cold environment since goats increase heat production to keep the body temperature, which results in a smaller amount of energy available for milk production (especially that DM intake did not increase and CT goat lost BW). Thompson and Thompson [14] reported that exposure of Saanen goats for two days to 0.5 to 1.0 °C with still or blown air increase heat production and the metabolic rate by 18% and 46%, respectively, compared to a thermoneutral environment (22 °C). Heat production is also increased by up to 55% in shorn Suffolk-cross ewes kept at 0 °C [31]. Furthermore, this decrease in milk yield could be related to decreased prolactin secretion. Tucker and Wettemann [32] reported that heifers exposed to cold temperatures had reduced blood concentrations of prolactin and growth hormone, and both hormones are important for lactation in goats [33].

The CT goats produced milk with greater (*p* < 0.05) contents of fat, protein, and lactose compared with TN goats, whereas milk SCC did not vary between treatments (Table 3). In agreement with our results, McBride and Christopherson [31] found that Suffolk-cross shorn ewes exposed to 0 °C produce milk with greater fat, protein, and lactose contents. Several studies have demonstrated that milk fat content increases when dairy cows are exposed to cold ambient temperatures [7,34]. In fact, mammary uptake of free fatty acids increased dramatically in Saanen goats exposed to cold ambient temperatures [35]. There was a tendency (*p* < 0.10) of treatment × period interaction for SCC due to the fact that SCC did not vary between TN and CT goats in period 1 (6.41 vs. 6.26, SED = 0.18, *p* = 0.440), but tended to increase by cold temperatures in period 2 (6.19 vs. 6.54, SED = 0.18, *p* = 0.084).

TN and CT goats yielded similar amounts of fat, protein, and lactose (Table 3). The fact that CT goats produced similar yields of milk components despite producing 13% less milk might indicate that the increment in the percentages of milk components in CT goats is partially caused by a concentration effect (CT goats drank less water and produced more concentrated milk). Consequently, energy-corrected milk was reduced by only 4% in CT goats, and this difference was not significant (*p* = 0.562) when compared to TN goats.

### 3.4. Blood Indicators

Values of the main blood metabolites in TN and CT goats are shown in Table 4. Compared to TN goats, CT goats experienced greater (*p* < 0.05) values of glucose and tended (*p* < 0.10) to have greater NEFA, but lower (*p* < 0.05) values of BHB and triglycerides. Blood levels of insulin and cholesterol did not vary between the TN and CT goats. In accordance with our glucose results, crossbred sheep exposed to 2 to 4 °C for 5 days have greater values of blood glucose concentration and turnover, regardless of the level of energy intake [36]. Additionally, exposure to a cold environment increases the output of glucose from the liver [37], and, consequently, glucose in the circulation might be increased [15]. This extra glucose in the circulation was not used for milk lactose since the milk lactose yield did not vary between TN and CT groups, but this glucose could have been used by other tissues (e.g., muscles) to increase heat production. Treatment × period interaction tended (*p* < 0.10) to be significant for blood glucose levels as TN goats had lower blood glucose than CT goats in period 1 (61.8 vs. 68.3 mg/dL, SED = 1.9, *p* < 0.01). Blood glucose in period 2 was also lower in TN than CT goats, but the difference was not significant (61.9 vs. 64.1 mg/dL, SED = 1.9, *p* = 0.343).

Blood glucose levels are mainly regulated by insulin, glucagon, growth hormone, and insulin sensitivity. Blood insulin concentration values did not vary between TN and CT goats (Table 4). Other hormones that are increased in cold temperatures, such as epinephrine [38], triiodothyronine [27], thyroxine [39], and cortisol [15] enhance glucose production and may synergistically result in increased blood glucose during cold exposure. In addition, concentration of blood glucagon is greater in cross-bred sheep during cold exposure (2 °C) than in a thermoneutral (20 °C) environment [26]. Although not measured in the present experiment, but might be supposed, this modified hormonal milieu could explain the increased blood glucose levels in CT goats.

Compared to TN goats, CT animals had similar DM intake and produced similar amounts of energy-corrected milk on average. Heat production is expected to increase in our CT goats as previously observed in Suffolk-cross sheep [31]. It is most likely that CT goats compensated for the extra energy needed for heat production by mobilizing body fat, which is shown by the tendency of greater (*p* < 0.10) blood NEFA concentrations (Table 4). In agreement with our results, heifers kept under 0 °C have greater basal levels of blood glucose and NEFA compared to heifers under 20 °C [40]. Cortisol levels in the blood of Saanen goats increased in the cold environment [15]. Additionally, Sasaki and Weekes [39] indicated that cold exposure increases catecholamine secretion through the enhanced activity of the hypothalamic-pituitary-adrenal system. Glucocorticoids are known to have lipolytic effects and favor the mobilization of body lipid tissue. However, Collier and Gebremedhin [41] reported that acute but not chronic heat stress activates the hypothalamic-pituitary axis and rapidly increases plasma cortisol in dairy cattle. This contradicts the fact that, at the end of prolonged cold exposure (blood samples were collected at day 21), blood NEFA tended to be greater in CT than TN goats (Table 4). This finding might indicate that the increased NEFA at week 3 of cold exposure in our CT goats was independent of the activation of the hypothalamic-pituitary axis. Since body lipid mobilization includes free fatty acids and glycerol, this glycerol could have been used in the liver to produce glucose [42], which may also partially explain the greater blood glucose level detected in CT goats (Table 4).

We speculate that CT goats increased the oxidation of mobilized free fatty acids to CO_2_ in the liver and also in skeletal muscles, not only to produce energy, but also to avoid both excessive fat accumulation in the liver (fatty liver) and the synthesis of hepatic ketone bodies. This assumption is supported by the fact that blood levels of BHB were lower (*p* < 0.05) in CT compared to TN goats (Table 4). Synthesis of NEFA in the liver could cause fat accumulation if the amounts of free fatty acids exceed the liver capacity to oxidate them to CO_2_ or ketone bodies. As a remedy, some triglycerides could be exported as very low-density lipoproteins (triglycerides and cholesterol). However, it seems that this did not occur in CT goats as blood triglycerides were lower (*p* < 0.01) in CT than TN goats, and blood cholesterol did not vary between groups (Table 4).

We cannot rule out the possibility that blood NEFA were partially taken up by the mammary gland for fat synthesis (although milk fat yield did not change) as commonly observed during the periods of negative EB in dairy cows [43]. Consequently, NEFA were not available for ketone body synthesis in the liver, which is an additional explanation of why CT goats had lower BHB levels (Table 4).

Blood values of pH, urea, K, total CO_2_, bicarbonate, base excess, and anion gap did not vary (*p* > 0.10) between TN and CT goats (Table 5). Nevertheless, lower values of Na (*p* < 0.05) and Cl (*p* < 0.10) were detected in CT goats compared to TN animals. Sodium and Cl are the major extracellular cation and anion, respectively. Changes in blood Cl tend to parallel those of Na because renal reabsorption of Na is accompanied by reabsorption of Cl [44]. The CT goats drank less water when compared to TN goats (Table 3) and it is possible that there was some degree of fluid decrease. Fluid decrease in ruminants is typically combined with loss of electrolytes [44], which might explain the reduced blood Na and Cl levels. Despite these reductions in blood Na and Cl, blood pH, total CO_2_, and anion gap values did not vary, which indicates that the metabolic acid-base status was not affected by cold temperatures in the current experiment. The decrease in blood Na and Cl concentrations by cold in the present study contrasts with what has been reported in heat-stressed goats where blood Cl concentration is increased by high ambient temperatures [28]. The values of partial pressure of CO_2_ tended to be greater (*p* < 0.10) in CT than in TN goats. This is consistent with the lower respiratory rate (Table 2) and less wash out of CO_2_ in CT goats. This result is the opposite of what happens in dairy goats under heat stress conditions [12,28], where heat-stressed goats experience less blood CO_2_ values due to accelerated respiration.

Hematocrit and hemoglobin tended to be more elevated (*p* < 0.10) in CT than TN goats, which might be related to the lower water consumption (Table 2). The increase in hematocrit in the current study agrees with results of lactating Saanen [14] and non-lactating Nubian [45] goats exposed to cold temperatures (0 to 1 °C).

At the end of the cold period, we observed that CT goats had thicker hair, possibly as a protection from the cold temperatures, but hair data were not recorded. Piloerection increases the external insulation. The rate of wool growth increases in Merino sheep exposed to 9 °C compared to 26 °C [27]. Furthermore, cattle in cold conditions reduce hair shedding and have a denser hair coat than cattle in warm conditions [46].

## 4. Conclusions

Dairy goats were sensitive to cold temperatures and responded to it with a reduced rectal temperature and respiratory rate. Goats exposed to cold temperatures produced less milk yield, but their milk was more concentrated. Consequently, yields of fat, protein, and energy-corrected milk were similar between TN and CT goats. The CT goats mobilized body fat tissue as indicated by the tendency of greater blood NEFA compared to TN goats. Blood insulin levels were similar, but CT goats were able to achieve greater blood glucose levels. The mobilized fatty acids together with the increased blood glucose could have been used in muscles for heat production under cold temperatures. Based on the obtained results, exposure of dairy goats to a temperature below zero should be avoided. However, more research is needed to test the effect of different cold intensities and durations in dairy goats.

## Figures and Tables

**Table 1:** Chemical composition and nutritive value (dry matter basis) of the ration.

Item	Total Mixed Ration
Component, %	
Dry matter	81.8
Organic matter	81.7
Crude protein	16.6
Neutral detergent fiber	33.5
Acid detergent fiber	23.2
Nutritive value ^1^	
UFL, ^2^/kg	0.85
NE_L_, ^3^ Mcal/kg	1.50
PDI, ^4^ g/kg	88.3
PDIA, ^5^ g/kg	40.2
RPB, ^6^ g/kg	31.2
Ca, g/kg	9.09
P, g/kg	2.91

^1^ Calculated according to the Institut National de la Recherche Agronomique [24]. ^2^ Net energy for lactation (1 UFL = 1.76 Mcal of NE_L_). ^3^ 1 UFL = 1.76 Mcal of NE_L_. ^4^ Protein digestible in the intestine from dietary and microbial origin. ^5^ Protein digestible in the intestine from dietary origin. ^6^ Rumen protein balance.

**Table 2:** Rectal temperature and respiratory rate measured at 8:00 a.m., 12:00 p.m., and 5:00 p.m. in dairy goats under thermoneutral (TN, 15 to 20 °C) or cold temperature (CT, −3 to 6 °C) conditions ^1^.

Item	Treatment			Effect ^3^ (*p<*)		
	TN (*n* = 8)	CT (*n* = 8)	SED ^2^	Trt	Per	Trt × Per
Rectal temperature, °C						
08:00 h	38.78 ^a,b^	37.70 ^b^	0.27	0.001	-	-
12:00 h	38.64 ^b^	37.85 ^b^	0.27	0.006	-	-
17:00 h	38.82 ^a^	38.58 ^a^	0.26	0.368	-	-
Average	38.75	38.04	0.26	0.011	0.040	0.806
Respiratory rate, breaths/min						
08:00 h	30	25	0.9	0.001	-	-
12:00 h	33	27	1.0	0.001	-	-
17:00 h	33	27	1.0	0.001	-	-
Average	32	27	0.8	0.001	0.107	0.293

^a,b^ Values within the same treatment (at 8:00 a.m., 12:00 p.m., and 5:00 p.m.) with different superscripts differ (*p* < 0.05). ^1^ Values are presented as least-square means. ^2^ Standard error of the difference. ^3^ Effects of treatment (Trt), period (Per), and their interaction (Trt × Per).

**Table 3:** Productive variables of dairy goats under thermoneutral (TN, 15 to 20 °C) or cold temperature (CT, −3 to 6 °C) conditions ^1^.

Item	Treatment		SED ^2^	Effect ^3^ (*p*<)		
	TN (*n* = 8)	CT (*n* = 8)		Trt	Per	Trt × Per
Body weight change, kg	2.54	−0.64	0.83	0.005	0.019	0.133
DM intake, kg/d	2.21	2.09	0.08	0.112	0.804	0.280
Water consumption, L/d	5.17	4.01	0.15	0.001	0.001	0.816
Water: DM intake ratio	2.37	1.99	0.15	0.012	0.001	0.753
Milk yield, kg/d	1.83	1.59	0.05	0.001	0.001	0.449
Energy-corrected milk, ^4^ kg/d	2.15	2.06	0.15	0.562	0.036	0.234
Energy balance, Mcal/d	0.34	0.16	0.05	0.004	0.030	0.989
Milk composition, %						
Fat	4.70	5.33	0.19	0.004	0.040	0.366
Protein	3.42	3.91	0.20	0.032	0.856	0.106
Lactose	4.57	4.75	0.08	0.034	0.080	0.071
Fat yield, g/d	85.6	84.9	6.6	0.923	0.018	0.565
Protein yield, g/d	61.9	61.8	3.5	0.983	0.072	0.004
Lactose yield g/d	83.7	75.5	7.1	0.278	0.312	0.605
Log somatic cell count	6.30	6.40	0.13	0.458	0.822	0.082

^1^ Values are presented as least-square means. ^2^ Standard error of the difference. ^3^ Effects of treatment (Trt), period (Per), and their interaction (Trt × Per). ^4^ 3.5% fat, 3.2% protein ECM = kg milk × 0.327 + kg fat × 12.95 + kg protein × 7. 2.

**Table 4:** Plasma insulin and metabolites measured in dairy goats under thermoneutral (TN, 15 to 20 °C) or cold temperature (CT, −3 to 6 °C) conditions ^1^.

Item	Treatment		SED ^2^	Effect ^3^ (*p*<)		
	TN (*n* = 8)	CT (*n* = 8)		Trt	Per	Trt × Per
Insulin, mg/L	0.330	0.320	0.087	0.912	0.458	0.842
Glucose, mg/dL	62.1	66.2	1.36	0.012	0.242	0.088
Non-esterified fatty acids, mmol/L	0.103	0.186	0.045	0.085	0.805	0.806
ß-hydroxybutyrate, mmol/L	0.582	0.386	0.068	0.014	0.889	0.958
Triglycerides, mg/dL	23.3	16.9	1.67	0.002	0.024	0.736
Cholesterol, mg/dL	88.7	79.3	8.12	0.270	0.029	0.534

^1^ Values are least-square means. ^2^ Standard error of the difference. ^3^ Effects of treatment (Trt), period (Per), and their interaction (Trt × Per).

**Table 5:** Blood metabolites and acid-base indicators in dairy goats under thermoneutral (TN, 15 to 20 °C) or cold temperature (CT, −3 to 6 °C) conditions ^1^.

Item	Treatment		SED ^2^	Effect ^3^ (*p*<)		
	TN (*n* = 8)	CT (*n* = 8)		Trt	Per	Trt × Per
pH	7.428	7.401	0.017	0.135	0.470	0.960
Urea, mg/dL	24.9	23.3	1.9	0.426	0.302	0.309
Na, mmol/L	144.9	143.4	0.7	0.049	0.093	0.999
K, mmol/L	3.74	3.79	0.26	0.848	0.843	0.634
Cl, mmol/L	105.4	104.0	0.74	0.088	0.869	0.415
Hematocrit, %	19.8	21.5	0.9	0.078	0.989	0.980
Hemoglobin, g/dL	6.74	7.31	0.31	0.086	0.874	0.937
Total CO_2_, mmol/L	28.4	28.5	1.0	0.906	0.416	0.461
HCO_3_, mmol/L	27.1	27.4	1.0	0.806	0.339	0.451
pCO_2_, mm of Hg	40.6	44.0	1.7	0.083	0.726	0.454
Anion gap, mmol/L	16.3	16.0	0.51	0.633	0.347	1.000
Base excess	2.50	2.64	1.14	0.915	0.252	0.595

^1^ Values are presented as least-square means. ^2^ Standard error of the difference. ^3^ Effects of treatment (Trt), period (Per), and their interaction (Trt × Per).
